# A Rare Combination: Cold Agglutinin Disease Followed by Waldenström Macroglobulinemia—A Case of Early Treatment Response

**DOI:** 10.3390/diagnostics15202654

**Published:** 2025-10-21

**Authors:** Anna Kozub, Aleksandra Nasiek, Natalia Bohun, Martyna Bednarczyk, Łukasz Sędek, Sebastian Grosicki

**Affiliations:** 1 Student Research Group at the Department of Cancer Prevention, Faculty of Public Health in Bytom, Medical University of Silesia, 40-055 Katowice, Poland; 2 Student Research Group at the Department of Microbiology and Immunology, Faculty of Medical Sciences in Zabrze, Medical University of Silesia, 40-055 Katowice, Poland; 3 Clinical Department of Haematology and Cancer Prevention, Independent Public Healthcare Institution, Municipal Hospital Complex in Chorzów, 41-500 Chorzów, Poland; 4 Department of Cancer Prevention, Faculty of Public Health in Bytom, Medical University of Silesia, 40-055 Katowice, Poland; 5 Department of Microbiology and Immunology, Faculty of Medical Sciences in Zabrze, Medical University of Silesia, 40-055 Katowice, Poland

**Keywords:** Waldenström macroglobulinemia, lymphoplasmacytic lymphoma, cold agglutinin disease, rituximab, bendamustine, anaemia

## Abstract

**Background and Clinical Significance:** Waldenström macroglobulinemia (WM) is a rare, indolent B-cell non-Hodgkin lymphoma, characterised by the presence of monoclonal immunoglobulin M (IgM) and lymphoplasmacytic infiltration of the bone marrow. It is often associated with various haematological and systemic disorders, including previous cold agglutinin disease (CAD), a condition where cold-sensitive antibodies lead to haemolysis. **Case Presentation:** A 55-year-old male patient was admitted to the Internal Diseases Ward with symptoms of weakness, reduced effort tolerance, and weight loss, along with life-threatening normoblastic anaemia (haemoglobin [Hb]: 3.90 g/dL). Initial blood tests raised suspicion of CAD due to the presence of multiple blood clots, as well as a decrease in lymphocyte and neutrophil counts. CAD was then confirmed by a cold agglutinin titre of 1:2000 and direct antiglobulin test ([DAT] 4+). Two weeks later, upon transfer to the Haematological Diseases Ward, further investigation revealed elevated IgM levels (up to 31.55 g/L). Additional diagnostic tests, including serum protein electrophoresis, imaging, multiparametric flow cytometry, and bone marrow biopsy, confirmed the diagnosis of WM. The *L265P MYD88* mutation test was positive. Treatment with intravenous rituximab was initiated, followed by bendamustine/rituximab (BR) therapy protocol as first-line treatment. After two cycles, the patient’s clinical condition and laboratory results significantly improved, with a marked reduction in IgM (<0.4 g/L). Hb levels steadily rose to 12.60 g/dL, eliminating the need for further blood transfusions. **Conclusions:** This case highlights the importance of recognising the coexistence of CAD and WM, which may present with overlapping clinical features, including life-threatening anaemia. Extensive diagnostics and prompt treatment with combination therapy can lead to effective clinical improvement.

## 1. Introduction

Waldenström macroglobulinemia (WM) is an incurable haematological neoplasm within the non-Hodgkin lymphomas, described as lymphoplasmacytic lymphoma in the bone marrow and concurrent monoclonal IgM gammopathy in the serum [1]. If dermal symptoms on lower extremities like petechiae, oedema, or the sense of burning are additionally present, the disease was previously called hypergammaglobulinemic purpura [2]. WM is considered a rare disease, with an estimated 1000 to 1500 adults in the United States starting treatment each year following diagnosis [3]. The median age at diagnosis is approximately 70 years, and the incidence is more frequent among men [4]. According to current recommendations, WM does not require active treatment during the asymptomatic course of disease [4,5]. Patients admitting a familial history of WM or lymphoplasmacytic lymphoma are said to achieve lower survival rates [5]. The assessment of the response to the therapy is based on a percentage of decline in the concentration of M paraprotein in serum [5].

Furthermore, it is said that the patients with chronic primary cold agglutinin disease (CAD) represent approximately 15–25% of all autoimmune haemolytic anaemia (AIHA) cases, with prevalence ranging from 14 to 33 patients per million in the United States [6,7]. CAD is characterised by increased levels of autoreactive monoclonal cold agglutinins, most often of IgM κ origin, that induce haemolysis at 3–4 °C, or at higher temperatures in cases of greater thermal amplitude [6,8]. Moreover, a diagnosis of CAD in the absence of symptoms is not considered an indication for treatment [9]. According to the National Comprehensive Cancer Network (NCCN) guidelines (Version 1.2025), CAD may also be one of the WM indicators, which include, i.e., amyloidosis, anaemia, cytopenia, cryoglobulinemia, neuropathy, organomegaly, or hyperviscosity [9].

Therefore, we present a patient initially diagnosed with CAD, followed shortly by a diagnosis of WM, who was admitted with a critically low Hb level. Following two cycles of bendamustine/rituximab (BR) therapy, the disease was well controlled, achieving a complete and sustained response in accordance with current guidelines.

## 2. Case Report

A 55-year-old male patient was admitted to the Internal Diseases Ward with life-threatening normoblastic anaemia (haemoglobin [Hb] 3.90 g/dL). He reported weakness, deterioration of effort tolerance, and approximately 6 kg weight loss in eight months. Moreover, this patient suffered from an upper respiratory tract infection with recurrent fever a month before hospitalisation. The concomitant diseases were atherosclerosis and osteoarthritis.

CAD was initially suspected during the cross-matching test, which revealed the presence of multiple blood clots. The diagnosis was later confirmed with a cold agglutinin titre of 1:2000. In addition, direct antiglobulin test (DAT) was strongly positive (4+). Comprehensive blood tests revealed several abnormalities, including deficiencies in calcium and folic acid, elevated lactate dehydrogenase (LDH) levels (up to 264 U/L), and increased immunoglobulin M (IgM) (up to 12.60 g/L). Furthermore, a decline in the C3 component of the complement system was noted (37.30 mg/dL), along with reduced levels of immunoglobulin G (IgG) and immunoglobulin A (IgA). Immunofixation analysis identified the presence of an IgM κ isotype. No evidence of antinuclear antibodies (ANA) or any subtypes (ANA3) was found. Additionally, no active viral infections were detected, including human immunodeficiency virus (HIV), hepatitis B virus (HBV), hepatitis C virus (HCV), cytomegalovirus (CMV), and Epstein–Barr virus (EBV). Imaging showed enlarged, single axillary, para-aortic, and left external iliac lymph nodes (approximately 12 mm), splenomegaly (137 × 55 × 149 mm), and hepatomegaly (up to 180 mm).

The patient was first administered a transfusion of 6 units of packed red blood cells (RBC) with a heater, and his Hb level elevated (8.10 g/dL). Furthermore, he was treated with one pulse of methylprednisolone 250 mg intravenously and three times 500 mg intravenously, then the steroid therapy was prescribed as prednisolone 30 mg orally daily.

**Figure 1:**
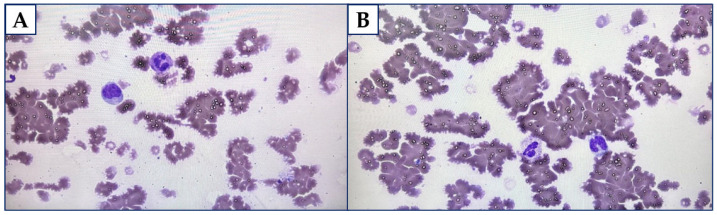
(**A**). The peripheral blood smear with the features of red blood cells (RBC) agglutination (total magnitude: 1000). (**B**). The peripheral blood smear with the features of RBC agglutination (total magnitude: 1000).

Two weeks following the CAD diagnosis, this patient was referred to the Haematology Ward for further diagnostics and treatment. He reported then a pharyngalgia. There was confirmed right axillary and inguinal lymphadenopathy (up to 15 mm) along with splenomegaly and hepatomegaly during physical examination. The blood test showed diminished levels of white blood cells (WBC) count (2.83∙10^3^/mm^3^), lymphocytes (0.41∙10^3^/mm^3^), and Hb (8.10 g/dL). The issue of agglutination led to false RBC count and other rates using that parameter. Furthermore, the level of IgM was stated as 31.55 g/L, and for β2-microglobulin it was 4.17 mg/L. The results of free light chain (FLC) assays were 56.85 mg/L for κ and 6.98 mg/L for λ. The ratio κ/λ was assessed as 8.145. Figure 1A,B display the peripheral blood smear with the features of RBC agglutination.

**Figure 2:**
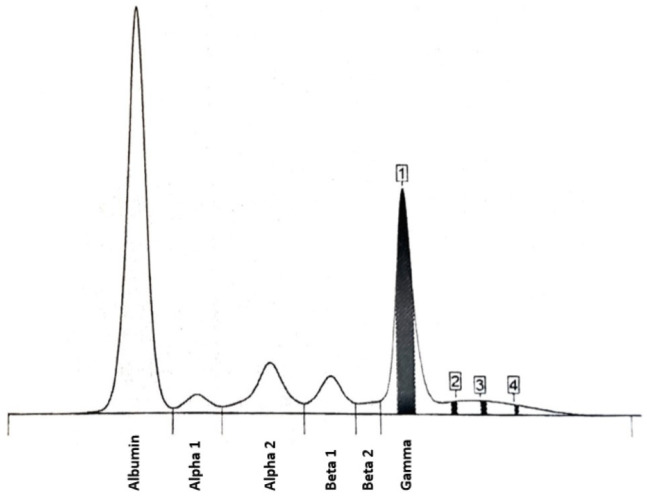
Serum protein electrophoresis (SPEP) results—protein fraction pattern.

Moreover, serum protein electrophoresis (SPEP) was performed (the results are displayed in Figure 2 and Table 1 in the main text and Table A1 in Appendix A.1). Peak 1—the level of M monoclonal protein (isotype IgM κ) was assessed as 1.31 g/dL (18.4%).

Multiparametric flow cytometry revealed a population of B-cell lineage characterised by SSC^low^, CD45^med/bright^, and CD19+ expression. These cells accounted for 5.9% of karyocytes and exhibited immunophenotypic features of differentiation. The list of used abbreviations and complete flow cytometry results (Table A2) may be found in Appendix A.2.

**Figure 3:**
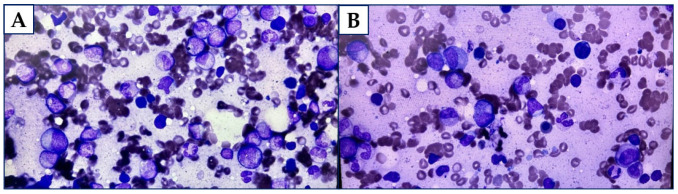
(**A**). Touch imprint on trephine biopsy (total magnitude: 400). (**B**). Touch imprint on trephine biopsy (total magnitude: 400).

The bone marrow trephine biopsy showed interstitial and tubercular lymphoid B-cell infiltrations (CD20+, PAX5+, CD23−/+ in a few cells, D1cyclin–, CD25−, CD10−, bcl6−, CD5−) and plasma cell infiltrations (CD138+, κ+, λ−), which were determined as approximately 40% of the total amount of the cells. There were also some reactive T lymphocytes, diffuse or located in tiny clusters, which accounted for 15% of the cells. The RBC line (CD71+) was relatively numerous, with the features of low dyserythropoiesis and some megaloblasts. The granulocyte (MPO+) and the megakaryocyte (CD42b+) lines were assessed as appropriate and with preserved maturation. The microscopic view suggested hyperglobulinaemic purpura. Figure 3A,B display touch imprints.

The man was tested for the *L265P MYD88* mutation with a positive result. The patient underwent the transfusion of 2 units of packed RBC with a heater. Moreover, he was treated with rituximab 700 mg intravenously in monotherapy. Methylprednisolone was administered as part of the supportive therapy. His clinical condition improved with WBC 3.26∙10^3^/mm^3^, Hb 9.30 g/dL, lymphocytes 0.22∙10^3^/mm^3^, total Ig 0.11 g/L. RBC count, as well as the other rates using the amount of RBC, could not be assessed due to agglutination. He has not required any blood transfusions since then.

**Figure 4:**
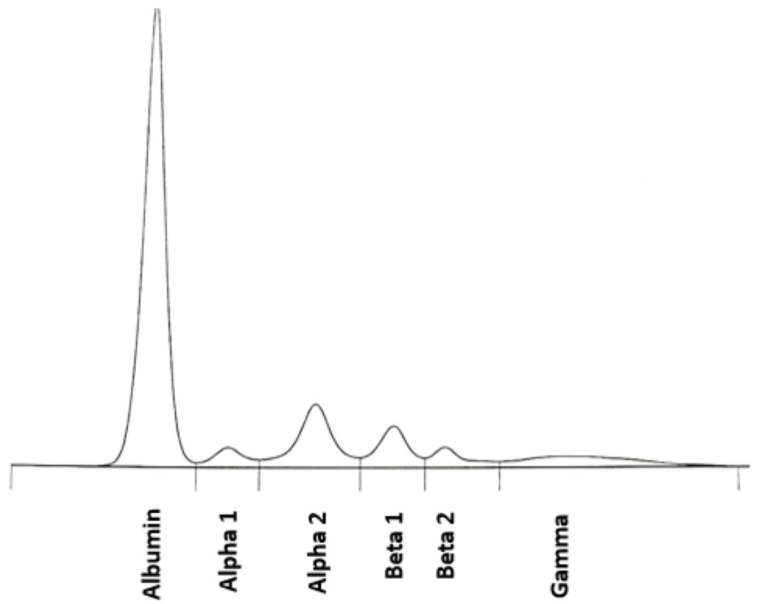
SPEP results—protein fraction pattern on completion of the second cycle of bendamustine/rituximab (BR) therapy.

The patient was subsequently administered combined cycles (each lasting 28 days) of BR therapy (rituximab: 375 mg/m^2^ on day 1, bendamustine: 90 mg/m^2^ during days 2–3). It was reported that his blood test results were WBC 2.95∙10^3^/mm^3^, Hb 12.60 g/dL, platelets level 146.00∙10^3^/mm^3^, lymphocytes 0.24∙10^3^/mm^3^ and neutrophils 2.85∙10^3^/mm^3^. The blood examination made after second cycle completion showed WBC 4.57∙10^3^/mm^3^, Hb 12.60 g/dL, platelets level 170.00∙10^3^/mm^3^, lymphocytes 0.25∙10^3^/mm^3^ and neutrophils 2.85∙10^3^/mm^3^. His IgM level was assessed as <0.4 g/L, and free κ chains as 6.61 mg/L. Figure 4 in the main text and Table A3 in Appendix A.3 present the outcomes of the SPEP examination.

After completing this primary stage of treatment, the patient was transferred to a hospital with a lower referral level closer to home. His overall clinical condition and the response to therapy have remained good.

## 3. Discussion

There are only a few case reports found on the issue of WM concealed due to cold AIHA. In this context, our case report raises the importance of awareness and further association of life-threatening anaemia with rare diseases, as well as further complex diagnostics and primary management. The first patient with concurrent WM and CAD disorders was described by Suzuki et al. in 1986 [10]. Hattori et al. reported the case of a patient with an initial Hb level of 4.09 g/dL. The amendment was observed in 4 weeks since the administration of rituximab, both in Hb level (8.7 g/dL), and in thermal amplitude (4096 in 4 °C compared to the titre of >32,768 at the diagnosis) [11]. However, such a treatment scheme turned out to be insufficient for a female with additional pancytopenia (baseline Hb 6.1 g/dL) despite an improvement in CAD management. Moreover, Caballero et al. admitted that she was diagnosed later with concurrent invasive fungal infection affecting the bone marrow, and the next line of treatment (ibrutinib) was planned [12]. Since the reported cases, including our patient, involve severe or life-threatening anaemia, it is noteworthy that such presentations may contrast with the typical findings in CAD or WM, where Hb levels are usually only slightly to moderately reduced. Moreover, given the limited data in the literature regarding the management of patients with concurrent CAD and WM, there is a clear need to raise and promote clinical vigilance among healthcare professionals.

In terms of diagnosis, the first signs of WM, often noticed by patients, include fatigue, lymphadenopathy, oedema, and petechiae, which are mainly located on the lower extremities [13]. To the contrary, our patient did not develop the aforementioned skin symptom. Broad-spectrum diagnostics towards concurrent viral disorders is also performed, because WM and CAD are associated with infections like HIV/AIDS, HCV, herpes zoster, or influenza [14]. In addition, the use of quantitative polymerase chain reaction and next-generation sequencing allows thorough examination of somatic mutations. Mutations like *MYD88* (>90% of all cases), *CXCR4* (~30% of all patients), *BIRC3*, *CD79B*, and hereditary issues support the development of WM in the majority of patients [15,16]. However, some studies showed that the *MYD88* mutation is absent or rare in cases of CAD [17,18]. Khwaja et al. showed that patients with combined WM and some IgM disorders may also develop later cryoglobulinemia, although it is often evaded in primary diagnosis [19]. According to the European Society For Medical Oncology (ESMO) guidelines (2018), in case of WM suspicion, other examinations may also be conducted, including cardiac troponins, N-terminal pro-B-type natriuretic peptide, von Willebrand factor, serum viscosity, 24 h urine protein collection, electromyography, anti-ganglioside M1 or myelin-associated globulin antibodies, depending on the clinical condition of the patient [20]. Some inflammatory or autoimmune diseases reported by patients or their relatives also increase the possibility of later WM diagnosis [15]. Thus, in the context of potentially non-haematological patients with suspected rheumatological, dermatological or infectious diseases presenting an unusual course, we suggest the inclusion of complex blood examinations during their diagnostics, according to the guidelines for WM. Furthermore, we finally confirmed WM in the following investigations: bone marrow biopsy, immunofixation, SPEP, and imaging, as these have become the gold standard in WM diagnostics. The concentrations of Hb (≤11.5 g/dL), platelets (≤100∙10^3^/mm^3^), IgM (>7 g/dL) or β_2_-microglobulin (>3 mg/L), followed by the most important factor: the age of the ill (>65 years), are crucial for further proper risk assessment, according to the International prognostic scoring system for Waldenström macroglobulinemia (ISSWM) [5]. Our patient belonged to the high-risk category.

Moreover, CAD is often associated not only with haematological diseases, including WM, but also with common viral and bacterial infections or autoimmune disorders [21]. Furthermore, the issue of frequent blood test monitoring due to possible changes over time should also be considered, as the analysis of our patient’s previous blood test results did not indicate CAD before the described hospitalisation. If CAD is considered, the thermal activity and titre (>64) are measured to confirm the diagnosis and whether the cold agglutinins could induce haemolysis [22,23]. The titre was calculated as 2000 in this case report. As presented to some extent in the case description, the blood test resulting in CAD diagnosis may include elevated levels of LDH, reticulocytes, and bilirubin, a positive outcome for C3d protein, and a negative result for IgG in DAT. Hb level is said to be usually mildly or moderately lowered (Hb >10 g/dL or approximately 8–10 g/dL); however, life-threatening anaemia may also occur, including our patient [23,24]. Furthermore, recurrent fever and such cutaneous symptoms as acrocyanosis, livedo reticularis, or even Raynaud’s phenomenon may occur in clinical examination due to thermal problems with the circulation system [24].

Nowadays, CAD treatment protocols have advanced due to extensive research into the aetiopathogenesis of CAD and the development of drugs targeting various points, such as B-cell, plasma cell, and complement-directed therapies [24,25]. What is more, according to the NCCN guidelines (Version 1.2025), preferred regimens for primary WM therapy include BR (category 2), ibrutinib ± rituximab (IR, category 1) or zanubrutinib (category 1). Other recommended schemes involve multiple combinations of drugs, also including, for instance, bortezomib dexamethasone carfilzomib ixazomib, cyclophosphamide, prednisone [9]. Furthermore, the complement-directed approach may include sutimlimab, rilibrupart, eculizumab, and pegcetacoplan [24,25]. However, the limitations of these novel drugs, such as temporary efficacy or the need to manage some untargeted adverse events, have been described [24,26]. Last, but not least, the continuous use of older, less effective treatments remains common worldwide [27]. Corticosteroid therapies may require high regular dosages, with remission achieved in fewer than 20% of patients. Consequently, high doses often cause serious adverse events, although their frequency in CAD is unknown [28].

The literature search identified several comparative analyses examining the clinical outcomes of various WM treatment protocols. Our patient received the BR scheme during his stay in the Haematology Ward due to its demonstrated efficacy as primary therapy. For instance, Paludo et al. conducted a comparative study on the clinical results of BR therapy (bendamustine: 90 mg/m^2^ on days 1 and 2; rituximab: 375 mg/m^2^ on day 1) in 60 patients and the dexamethasone/rituximab/cyclophosphamide (DRC) scheme (dexamethasone 20 mg on day 1, rituximab 375 mg/m^2^ on day 1, and cyclophosphamide 100 mg/m^2^ on days 1–5) in 100 patients [29]. For the primary treatment subgroup, the 2-year progression-free survival (PFS) for BR (43 patients) was 88.0%, compared with 61.0% for the 50 patients treated with the DRC (*p* = 0.07). The presence of the *MYD88* mutation did not correlate with the outcomes observed [29,30]. Moreover, comparable, very satisfactory results after both short and extended follow-up periods were confirmed in the multicentre analysis of 69 WM patients by the French Innovative Leukaemia Organisation (FILO) [31,32]. Nearly all patients exhibited some level of response after primary BR treatment (bendamustine: 90 mg/m^2^ on days 1–2, rituximab 375 mg/m^2^ on day 1), with the cumulative incidence of an overall response approximately 69.6% at 3 months, rising to 91% by 6 months. However, no association with mutational status was observed [31]. The median time to the start of second-line treatment due to relapse was 35.3 months [32].

To conclude the issue of BR protocol, in the context of our case, the meta-analysis based on seven phase II trials and four phase III trials by Chan et al. indicated that BR scheme-treated patients achieved a pooled response rate of 46%, when compared to 26% in patients after IR treatment, and 33% assessed in the group with administered bortezomib/dexamethasone/rituximab/cyclophosphamide regimens [33]. Similarly, the pooled 2-year PFS also favoured BR over other schemes, with rates of 89%, 82%, and 81%, respectively [33].

The limitation of this case report is the short follow-up period, as the patient was transferred to another medical centre, resulting in only the basic panel of confirmed mutations. Nowadays, as more research depends on groups of patients with specifically examined genetics, practitioners should also remember to thoroughly perform such testing. Moreover, due to the retrospective nature of the study and the patient’s previous medical history outside our hospital, it was impossible to conduct an advanced analysis of prior blood test results. However, we believe that the combination of such rare disorders and the significant, rapid improvement in the patient’s overall condition before and shortly after chemoimmunotherapy administration is noteworthy, especially for young practitioners. Currently published studies lack a specific analysis of clinical outcomes from patients with both CAD and WM.

## 4. Conclusions

The concurrent presence of CAD can obscure the diagnosis of WM, as overlapping clinical features may complicate the identification of either condition. A thorough clinical evaluation is essential to identify coexisting haematological disorders, particularly when their manifestations—such as anaemia, fatigue, or hyperviscosity—may be nonspecific or mutually masking. Advanced diagnostic tools, including comprehensive blood panels, immunophenotyping, genetic profiling, and bone marrow biopsy, are critical for establishing an accurate diagnosis of WM in the context of CAD. The combination of rituximab and bendamustine has demonstrated therapeutic efficacy, often leading to rapid and meaningful clinical improvement in patients affected by both WM and CAD. Furthermore, the issue of potentially more complex haematological background should also be considered while diagnosing patients with suspected other internal, rheumatological, and infectious diseases, to avoid potentially asymptomatic or oligosymptomatic life-threatening anaemia.

## Figures and Tables

**Table 1:** SPEP results—protein fractions.

Protein Fraction	Result [%]	Reference [%]	Result [g/dL]	Reference [g/dL]
Albumin	48.4	54.3–65.5	3.44	3.80–4.60
α1	2.9	1.2–3.3	0.21	0.08–0.23
α2	9.7	8.3–15.0	0.69	0.58–1.10
β1	5.9	6.5–11.5	0.42	0.46–0.81
β2	1.5	2.5–7.2	0.11	0.18–0.50
γ	31.6	7.1–19.5	2.24	0.50–1.40

## Data Availability

The original contributions presented in this study are included in the article. Further inquiries can be directed to the corresponding author.

## Abbreviations

The following abbreviations are used in this manuscript:

WM	Waldenström macroglobulinemia
IgM	Immunoglobulin M
CAD	Cold agglutinin disease
Hb	Haemoglobin
DAT	Direct antiglobulin test
AIHA	Autoimmune haemolytic anaemia
NCCN	National Comprehensive Cancer Network
BR	Bendamustine/rituximab
LDH	Lactate dehydrogenase
IgG	Immunoglobulin G
IgA	Immunoglobulin A
ANA	Antinuclear antibodies
HIV	Human immunodeficiency virus
HBV	Hepatitis B virus
HCV	Hepatitis C virus
CMV	Cytomegalovirus
EBV	Epstein–Barr virus
RBC	Red blood cells
WBC	White blood cells
FLC	Free light chain
SPEP	Serum protein electrophoresis
ESMO	European Society For Medical Oncology
ISSWM	International prognostic scoring system for Waldenström macroglobulinemia
DRC	Dexamethasone/rituximab/cyclophosphamide
PFS	Progression-free survival
IR	Ibrutinib/rituximab
FILO	French Innovative Leukaemia Organisation

## Appendix A

### Appendix A.1. SPEP Results During Diagnostics

**Table A1:** SPEP results during diagnostics—peaks.

Peak	Result [%]	Result [g/dL]
1	18.4	1.31
2	0.6	0.04
3	0.6	0.04
4	0.3	0.02

### Appendix A.2. Complete Flow Cytometry Examination

**Table A2:** Immunophenotypical features of dominant B-cell line origin SSC^low^ CD45^med/bright^ CD19+.

Immunophenotype	Result	Additional Remarks
CD5	-	
CD23	-	
CD20	+ (bright)	
CD79b	+ ~83%	
CD22	+	
CD10	-	
CD38	−/+ ~47% (part pos)	
CD200	+/− ~74% (het)	
CD103	-	
CD71	−/+ ~43%	
κ	+ ~85%	Interpretation of this result was interrupted due to nonspecific reactions of the antibodies.
λ	~1%	
CD34	-	
jTdT	-	
CD138	−/+ ~22%	
CD3	-	
CD7	-	
CD8	-	
CD4	-	
CD56/CD16	-	
CD117	-	

Furthermore, there were also other cell lines detected in this examination:

- blast cells from myeloid and monoidal lines SSC^med^ CD45^dim/med^ CD34−/+^part pos^ CD117+ HLA-DR+ CD33+^het^ CD64−/+ CD66c− CD14− (in total 2.2% of all cells, among them blast cells CD34+ were calculated as 0.3%),
- cells from monoidal line SSC^med^ CD45^med/bridght^ CD34− CD117− HLA−DR+^het^ CD64+^bright^ CD33+^bridght^ (3.6% of all cells, the majority of them (2.2% of all cells) were mature/maturing CD14+),
- cells from granulocyte line SSC^high^ CD45^med^ CD34− CD117− CD66c+ CD33+ (73.0% of all cells, the mature cells CD10+ accounted for 20% of cells from this line),
- promyelocytes SSC^high^ CD45^med^ CD34− CD117+ CD66c+ CD33+ (1.5% of all cells),
- plasma cells line SSC^med^ CD45^med/bright^ CD117− CD138+^bright^ CD38+^bright^ CD19+/− CD20- (ratio cytκ/cytλ = 57.6%/42.5% = 1.4; <0.1% of all cells, the cells did not show clearly monoclonal features in the assessment based on FLC κ and λ),
- T lymphocytes with dominant immunophenotype SSC^low^ CD45^bright^ CD3+ CD7+ CD5+ (5.3% of all cells):
  T lymphocytes SSC^low^ CD45^bright^ CD3+ CD4+ (2.0% of all cells),
  T lymphocytes SSC^low^ CD45^bright^ CD3+ CD8+ (2.8% of all cells),
  CD3+CD4+/CD3+CD8+ = 0.7
  natural killers T (NKT) cells SSC^low^ CD45^bright^ CD3+ CD5616+ (2.1% of all cells),
- natural killers (NK) cells SSC^low^ CD45^bright^ CD3− CD7+ CD56/CD16+ (0.9% of all cells).

**The list and explanation of symbols or abbreviations used in flow cytometry examination:**

❖ − or negative—<10% of positive cells in the population,
❖ −/+—10–50% of positive cells in the population,
❖ +/−—50–80% of positive cells in the population,
❖ + or positive—>80% of positive cells in the population,
❖ dim—the population is partly or entirely displaced towards the positive cells’ side, their expression is weaker in comparison with the positive cells,
❖ bright—homogenous population with only positive cells, which do not coincide with negative cells, their expression is the same or stronger in comparison with the referential positive cells,
❖ het—heterogeneous expression and >50z5 positive cells,
❖ part pos—there are two populations of the cells detected, only one of them is positive (≥10% positive cells),
❖ low, med, high—the characteristics of parameters of FSC and SSC scattering,
❖ neg, dim, med, bright—the characteristics of CD45 expression.

### Appendix A.3. SPEP Results During the Treatment

**Table A3:** SPEP results—protein fractions on completion of the second cycle of BR therapy.

Protein Fraction	Result [%]	Reference [%]	Result [g/dL]	Reference [g/dL]
Albumin	62.3	54.3–65.5	3.92	3.80–4.60
α1	3.9	1.2–3.3	0.25	0.08–0.23
α2	14.0	8.3–15.0	0.88	0.58–1.10
β1	7.8	6.5–11.5	0.49	0.46–0.81
β2	4.2	2.5–7.2	0.26	0.18–0.50
γ	7.8	7.1–19.5	0.49	0.50–1.40

## References

[1] Swerdlow S.H., Campo E., Harris N.L., Jaffe E.S., Pileri S.A., Stein H., Thiele J., Vardiman J.W. (2008). World Health Organization Classification of Tumours of Haematopoietic and Lymphoid Tissues.

[2] Finder K.A., McCollough M.L., Dixon S.L., Majka A.J., Jaremko W. (1990). Hypergammaglobulinemic Purpura of Waldenström. J. Am. Acad. Dermatol..

[3] Sekhar J., Sanfilippo K., Zhang Q., Trinkaus K., Vij R., Morgensztern D. (2012). Waldenström Macroglobulinemia: A Surveillance, Epidemiology, and End Results Database Review from 1988 to 2005. Leuk. Lymphoma.

[4] Ghafoor B., Masthan S.S., Hameed M., Akhtar H.H., Khalid A., Ghafoor S., Allah H.M., Arshad M.M., Iqbal I., Iftikhar A. (2024). Waldenström Macroglobulinemia: A Review of Pathogenesis, Current Treatment, and Future Prospects. Ann. Hematol..

[5] Gertz M.A. (2025). Waldenström Macroglobulinemia: 2025 Update on Diagnosis, Risk Stratification, and Management. Am. J. Hematol..

[6] Berentsen S., Röth A., Randen U., Jilma B., Tjønnfjord G.E. (2019). Cold Agglutinin Disease: Current Challenges and Future Prospects. J. Blood Med..

[7] Bozzi S., Umarje S., Hawaldar K., Tyma J., Schinkel J., Agatep B., Pulungan Z., Petruski-Ivleva N. (2023). Prevalence and Incidence of Primary Autoimmune Hemolytic Anemia and Cold Agglutinin Disease in the United States, 2016-2022: A Retrospective Study in Administrative Claims. Blood.

[8] Ulvestad E., Berentsen S., Bø K., Shammas F.V. (1999). Clinical Immunology of Chronic Cold Agglutinin Disease. Eur. J. Haematol..

[9] NCCN Clinical Practice Guidelines in Oncology (NCCN Guidelines ^®^) Waldenström Macroglobulinemia/Lymphoplasmacytic Lymphoma. Version 1.2025—13 September 2024. https://www.nccn.org/professionals/physician_gls/pdf/waldenstroms.pdf.

[10] Suzuki M., Noda T., Kodama H., Sahashi K., Wakita A. (1986). A case of Waldenström’s macroglobulinemia with cold agglutinin disease. Rinsho Ketsueki.

[11] Hattori N., Ishii N., Ariizumi H., Adachi D., Matsuda I., Nakamaki T., Tomoyasu S. (2010). Improvement of the Thermal Amplitude after Rituximab Treatment for Cold Agglutinin Disease with Waldenström’s Macroglobulinemia. Ann. Hematol..

[12] Caballero J.C., Askari E., Carrasco N., Piris M.A., Perez de Camino B., Pardo L., Cornago J., Lopez-Lorenzo J.L., Llamas P., Solan L. (2023). Invasive Cutaneous Candidiasis, Autoimmune Hemolytic Anemia and Pancytopenia: A Challenging Scenario for Waldenström Macroglobulinemia in an Elderly Patient. Biomedicines.

[13] Theisen E., Lee D.E., Pei S., Schauder D.M., Chiu Y.E., Brandling-Bennett H., Curran M.L., Klein-Gitelman M., Co D.O., Arkin L.M. (2020). Hypergammaglobulinemic Purpura of Waldenström in Children. Pediatr. Dermatol..

[14] Kristinsson S.Y., Koshiol J., Björkholm M., Goldin L.R., McMaster M.L., Turesson I., Landgren O. (2010). Immune-Related and Inflammatory Conditions and Risk of Lymphoplasmacytic Lymphoma or Waldenstrom Macroglobulinemia. J. Natl. Cancer Inst..

[15] Manasanch E.E., Kristinsson S.Y., Landgren O. (2013). Etiology of Waldenström Macroglobulinemia: Genetic Factors and Immune-Related Conditions. Clin. Lymphoma Myeloma Leuk..

[16] Østergaard S., Schejbel L., Breinholt M.F., Pedersen M.Ø., Hammer T., Munksgaard L., Nørgaard P., Høgdall E., Gjerdrum L.M.R., Nielsen T.H. (2024). Mutational Landscape in Waldenström Macroglobulinemia Evaluated Using a Next-Generation Sequencing Lymphoma Panel in Routine Clinical Practice. Leuk. Lymphoma.

[17] de Tute R., Rawstron A., Evans P., Owen R. (2015). Cold Agglutinin Disease Is a Phenotypically Distinct Clonal B-Cell Disorder. Clin. Lymphoma Myeloma Leuk..

[18] Cao X.X., Meng Q., Cai H., He T.H., Zhang C.L., Su W., Sun J., Li Y., Xu W., Zhou D. (2017). Detection of MYD88 L265P and WHIM-like CXCR4 Mutation in Patients with IgM Monoclonal Gammopathy Related Disease. Ann. Hematol..

[19] Khwaja J., Vos J.M.I., Pluimers T.E., Japzon N., Patel A., Salter S., Kwakernaak A.J., Gupta R., Rismani A., Kyriakou C. (2024). Clinical and Clonal Characteristics of Monoclonal Immunoglobulin M-Associated Type I Cryoglobulinaemia. Br. J. Haematol..

[20] Kastritis E., Leblond V., Dimopoulos M.A., Kimby E., Staber P., Kersten M.J., Tedeschi A., Buske C. (2018). Waldenström’s macroglobulinaemia: ESMO Clinical Practice Guidelines for diagnosis, treatment and follow-up. Ann. Oncol..

[21] Barcellini W., Fattizzo B. (2020). The Changing Landscape of Autoimmune Hemolytic Anemia. Front. Immunol..

[22] Joly F., Schmitt L.A., Watson P.A.M.G., Pain E., Testa D. (2022). The Burden of Cold Agglutinin Disease on Patients’ Daily Life: Web-Based Cross-Sectional Survey of 50 American Patients. JMIR Form. Res..

[23] Swiecicki P.L., Hegerova L.T., Gertz M.A. (2013). Cold Agglutinin Disease. Blood.

[24] Berentsen S., Fattizzo B., Barcellini W. (2023). The Choice of New Treatments in Autoimmune Hemolytic Anemia: How to Pick from the Basket?. Front. Immunol..

[25] Barcellini W., Fattizzo B. (2024). The Evolving Management Algorithm for the Patient with Newly Diagnosed Cold Agglutinin Disease. Expert. Rev. Hematol..

[26] Berentsen S., Barcellini W., D’Sa S., Randen U., Tvedt T.H.A., Fattizzo B., Haukås E., Kell M., Brudevold R., Dahm A.E.A. (2020). Cold Agglutinin Disease Revisited: A Multinational, Observational Study of 232 Patients. Blood.

[27] Berentsen S. (2021). How I Treat Cold Agglutinin Disease. Blood.

[28] Nakasone H., Kako S., Endo H., Ito A., Sato M., Terasako K., Okuda S., Tanaka Y., Yamazaki R., Oshima K. (2009). Diabetes Mellitus Is Associated with High Early-Mortality and Poor Prognosis in Patients with Autoimmune Hemolytic Anemia. Hematology.

[29] Paludo J., Abeykoon J.P., Shreders A., Ansell S.M., Kumar S., Ailawadhi S., King R.L., Koehler A.B., Reeder C.B., Buadi F.K. (2018). Bendamustine and Rituximab (BR) versus Dexamethasone, Rituximab, and Cyclophosphamide (DRC) in Patients with Waldenström Macroglobulinemia. Ann. Hematol..

[30] Arulogun S.O., Brian D., Goradia H., Cooney A., Menne T., Koo R., O’Neill A.T., Vos J.M.I., Pratt G., Turner D. (2023). Bendamustine plus Rituximab for the Treatment of Waldenström Macroglobulinemia: Patient Outcomes and Impact of Bendamustine Dosing. Am. J. Hematol..

[31] Laribi K., Poulain S., Willems L., Merabet F., Le Calloch R., Eveillard J.R., Herbaux C., Roos-Weil D., Chaoui D., Roussel X. (2019). Bendamustine plus Rituximab in Newly-Diagnosed Waldenström Macroglobulinaemia Patients. A Study on Behalf of the French Innovative Leukaemia Organization (FILO). Br. J. Haematol..

[32] Laribi K., Poulain S., Willems L., Merabet F., Herbaux C., Roos-Weil D., Laribi de Materre I., Roussel X., Nudel M., Tricot S. (2024). Long-Term Results of Waldenström Macroglobulinaemia Treatment by Bendamustine and Rituximab: A Study on Behalf of the French Innovative Leukemia Organization (FILO). Br. J. Haematol..

[33] Chan W.-L., Chong V.C.L., Wee I.J.Y., Poon L.M., Chan E.H.L., Lee J., Chee Y.-L., Jeyasekharan A.D., Chng W.-J., Samuel M. (2023). Efficacy and Safety of Front-Line Treatment Regimens for Waldenstrom Macroglobulinaemia: A Systematic Review and Meta-Analysis. Blood Cancer J..

